# Immunoassay for pyruvate kinase M1/2 as an Alzheimer’s biomarker in CSF

**DOI:** 10.1515/biol-2025-1101

**Published:** 2025-07-07

**Authors:** Matthijs B. de Geus, Pia Kivisäkk, Bianca A. Trombetta, Betty M. Tijms, Pieter Jelle Visser, Steven E. Arnold, Becky C. Carlyle

**Affiliations:** Massachusetts General Hospital & Harvard Medical School, Department of Neurology, Boston, MA, United States of America; Alzheimer Center Amsterdam, Department of Neurology, Amsterdam Neuroscience, Vrije Universiteit Amsterdam, Amsterdam UMC, Amsterdam, The Netherlands; Department of Physiology, Anatomy & Genetics, University of Oxford, Oxford, United Kingdom; Kavli Institute for Nanoscience Discovery, University of Oxford, Oxford, United Kingdom

**Keywords:** Alzheimer’s disease, pyruvate kinase, biomarker, glucose metabolism, immunoassay

## Abstract

Alzheimer’s disease (AD) is characterized by amyloid-beta plaques and tau tangles in the brain, but these markers alone do not predict disease progression. The intersection of these pathologies with other processes including metabolic changes may contribute to disease progression. Brain glucose metabolism changes are among the earliest detectable events in AD. Pyruvate kinase (PKM) has been implicated as a potential biomarker to track these metabolic changes. We have developed an enzyme-linked immunosorbent assay (ELISA) to assess PKM levels in cerebrospinal fluid (CSF). First, we verified the relationship of CSF PKM levels with cognitive decline, revealing a correlation between elevated CSF PKM levels and accelerated cognitive decline in preclinical AD patients in a tau-dependent manner. We developed the ELISA using two PKM-specific antibodies and validated it through quality control steps, indicating robust quantification of PKM. We showed that ELISA measurements of PKM correlate with mass spectrometry values in matching samples. When tested on an independent cohort, the assay confirmed elevation of PKM in AD. These findings support the use of PKM as a potential biomarker for tracking early metabolic changes in AD, offering a novel tool for investigating metabolic alterations and their intersection with other underlying pathologies in AD progression.

## 1 Introduction

Alzheimer’s disease (AD) is a progressive neurodegenerative disorder, characterized by the accumulation of amyloid-beta plaques and tau tangles in the brain. Beyond these markers, there is significant heterogeneity in the progression of the cognitive decline amongst patient populations due to various underlying pathophysiologic contributions, such as metabolic disturbances, immune response, synaptic dysfunction, and vascular contributions [1–3]. These various underlying pathologies likely occur at different stages during disease development and progression, contributing to heterogeneity in cognitive decline [4]. With recent advances in amyloid targeting interventions for AD [5], it has become increasingly important to develop markers of these underlying processes to provide insights into the metabolic, immune, and vascular alterations that may diversely drive disease progression in different patient subgroups and at different stages of disease progression.

One of the earliest observed changes in AD is a shift in brain energy metabolism [6]. In the brain, a reduced uptake of glucose in the hippocampal and posterior cingulate regions can be measured through 18F-fluorodeoxyglucose-positron emission tomography (FDG-PET) in the early stages of AD [7–9]. In CSF, studies have consistently shown multiple enzymes of the glycolytic metabolism cascade, the primary pathway of glucose metabolism, to be upregulated in AD patients [10–13]. One recent study specifically indicated that glycolytic enzymes are only upregulated in patients that have both amyloid plaque and neurofibrillary tangle pathology, indicating a potential link between dysregulated glycolytic metabolism and tau pathology [14]. It remains unclear how the changes in glycolytic metabolism are associated with other pathological changes over the course of disease progression.

Pyruvate kinase (PKM) is a rate-limiting enzyme that converts phosphoenolpyruvate into pyruvate as the last step of glycolysis, a key step in cellular energy metabolism controlling the production of ATP [15]. Multiple studies have suggested a role for PKM in the glycolytic shift seen in AD [10,13,16]. A study on autosomal dominant AD indicated that PKM is one of the earliest markers to be elevated in the course of disease onset [4]. Studies in post-mortem brain tissue have also shown upregulation of glycolytic enzymes [17,18]. One study, focusing on the synaptic proteome, found an upregulation of glycolysis in people with dementia but not in resilient individuals who have significant amyloid deposition but no cognitive impairment [18]. A further study, which applied both an induced neuronal model of AD and analyzed post-mortem AD brain tissue, also indicated upregulation of glycolytic metabolism in both matrices, and suggested the potential for different PKM isoforms to enact a shift from aerobic to anaerobic glycolysis [19].

Crucial to the development of novel biomarkers for AD is the reliable detection with sufficient specificity and sensitivity. While untargeted mass spectrometry methods can identify novel potential biomarkers, they provide only relative quantification, requiring normalization and making cross-study comparisons challenging. In contrast, targeted immunoassays, such as ELISA, offer absolute quantification against a standard curve, facilitating comparability across studies when appropriately designed. Moreover, immunoassays are generally more accessible and facilitate clinical implementation where immunoassay-based platforms remain the standard for biomarker assessment [20].

In this study, we established an immunoassay that can be used in the future to track CSF PKM levels over the course of AD progression. First, to further establish the relevance of PKM as a marker of the early glycolytic shift in AD, we analyzed the relationship of CSF PKM levels with cognitive decline in a proteomics dataset previously published by Tijms et al. [2]. These data indicated that PKM levels are upregulated only in patients that are positive for both amyloid and tau pathology. Additionally, it was found that patients with preclinical AD and higher CSF PKM levels at baseline showed an accelerated cognitive decline over time. To further explore the potential of PKM as a biomarker for the changes in glucose metabolism that occur over the course of AD progression, we developed a Meso Scale Discovery (MSD)-based ELISA specifically designed to measure PKM levels in CSF. We validated the robustness of this assay through multiple quality control steps. The assay showed a strong correlation with PKM abundance obtained via mass spectrometry in matching samples. When applied to an independent validation sample set, the assay confirmed an upregulation of CSF PKM levels in patients who were positive for both amyloid and tau. Together, this work presents a novel assay to measure PKM levels in CSF and supports its use to track the glycolytic changes in the early stages of AD.

## 2 Methods

### 2.1 CSF sample collection

All CSF samples were collected through lumbar puncture after written informed consent at Massachusetts General Hospital by trained clinical staff, following standardized collection and processing protocols (IRB 2015P000221 and 2018P001989). All methods were performed in accordance with the ethical standards of the Declaration of Helsinki. Participants included patients that were clinically diagnosed with mild-cognitive impairment (MCI) or dementia due to AD and healthy controls. Diagnosis was established by an experienced neurologist (S.E.A.), based on symptom history, diagnoses of treating neurologist, laboratory data, neuroimaging, and neuropsychological testing, as available. Healthy controls had no clinical indication for neurodegenerative disease and had an average montreal cognitive assessment score of 26.8. Samples were collected between 8AM and 1PM. CSF levels of A-beta_1–40_, A-beta_1–42_, and ptau-181 were measured by commercially available ELISA assays either from Euroimmun (Lubeck, Germany) or ADmark (Athena diagnostics, Marlborough MA, USA). Samples that were measured with Euroimmun were classified as amyloid/tau (AT) positive based on AD biomarkers showing A-beta_42/40_ ratio below the in-house threshold of 0.0818, and the p-tau levels above the in-house threshold of 41.8. At these levels sensitivity is 91.6% and specificity is 91.3%. Samples assessed with ADmark were defined as AT positive based on an ATI score below 0.8 and a p-tau level higher than 61 pg/mL [21].


**Informed consent:** Informed consent has been obtained from all individuals included in this study.
**Ethical approval:** The research related to human use has been complied with all the relevant national regulations, institutional policies, and in accordance with the tenets of the Helsinki Declaration, and has been approved by the authors’ institutional review board or equivalent committee.

### 2.2 Mass spectrometry protein quantification

Mass spectrometry proteomics was performed using data-independent acquisition mass spectrometry as described in a previous study [10]. In short, total protein concentrations were normalized across all samples. Proteins were digested with LysC and trypsin. Data-Independent Acquisition liquid chromatography-mass spectrometry was performed in batches, including technical controls to ensure consistency. Protein identification and quantification were carried out using DIA-NN with an *in silico* generated library from the Uniprot Reference Homo Sapiens database (UP000005640). This process identified 576 unique protein groups expressed in at least 80% of samples. Batch effects were corrected using the ComBat algorithm. For the analysis here, PKM abundances from the samples tested with ELISA were log-transformed, *z*-scored, and scaled between 0 and 1.

### 2.3 Development of PKM ELISA

A sandwich ELISA assay was developed on the MSD platform to specifically quantify PKM in CSF. Two PKM rabbit monoclonal antibodies, C103A3 (Cell Signaling Technology, #3190) and C5E6 (Cell Signaling Technology, #3106), were obtained in carrier-free solution, and stored at −20°C. These antibodies bind two different epitopes on PKM and are not isoform specific, thus should bind both PKM1 and PKM2 isoforms. Antibody buffers were exchanged to MSD conjugation buffer (MSD, #R31AA) using Zeba spin columns (ThermoFisher, #87766) following standard manufacturer procedure. C5E6 was biotinylated (C5E6-BT) for use as capture antibody using EZ-Link Sulfo-NHS-LC-Biotin, No-Weigh Format (ThermoFisher, #A39257), following standard manufacturer’s procedure with a 10-fold molar excess of biotin. C103A3 was conjugated with MSD GOLD SULFO-TAG NHS-Ester (MSD, #R31AA) for use as detection antibody (C103A3-ST) following the manufacturer’s guidelines at a 20-fold molar excess of SULFO-TAG reagent. Conjugations were performed at 23°C for 2 h and conjugated antibodies were stored in MSD conjugation storage buffer (MSD, #R31AA).

### 2.4 Measurement of PKM in CSF with MSD ELISA

Levels of PKM in CSF were measured using our in-house developed MSD assay. The assay was conducted using streptavidin-coated small spot plates (MSD, #L45SA). Prior to the assay, the plates were blocked with 150 µL per well of MSD Blocker A (MSD, #R93BA). Plates were incubated for 1 h at room temperature with shaking at 700–800 rpm. After blocking, plates were washed three times with 150 µL per well of 1× PBS-T wash buffer. For the capture step, the capture antibody C5E6-BT was prepared in Diluent 100 (MSD, #R50AA) at a final concentration of 0.5 µg/mL. Capture antibody was added to the wells at 50 µL/well and incubated for 1 h at room temperature with shaking. Following incubation, plates were washed three times with wash buffer. Calibrators were prepared using recombinant PKM protein (Fisher Scientific, #7244PK020), which was serially diluted in Diluent 100 to create an 8-point calibration curve ranging from 1,000 to 4.1 ng/mL. All samples were diluted 3× in Diluent 100. Samples and calibrators were then added to the plate in duplicate, with 50 µL per well, and incubated for 1 h at room temperature with shaking. For detection, the detector antibody C10383-ST was prepared in Diluent 100 at a final concentration of 1 µg/mL. After washing the plates three times, 50 µL of detection antibody solution was added to each well and incubated for 1 h at room temperature with shaking. The plates were then washed a final time and read on the MESO QuickPlex SQ 120MM machine using 150 µL per well of 2× MSD Read Buffer T (MSD, #R92TC), diluted 4× in dH_2_O. On each plate, four plate-to-plate pooled CSF control samples were run to adjust for interplate variability.

### 2.5 Data analysis

Raw data as measured with the MESO QuickPlex were analyzed with Discovery Workbench 4.0.12 and further analyzed with R studio version 4.3.1. Coefficients of variation (CVs) were calculated between all sample duplicates and samples with CVs over 25% were excluded from downstream analysis. Raw data were adjusted for interplate variability by calculating an adjustment factor based on the plate-to-plate controls. Calculated concentrations that fell below lower limit of quantification (LLOQ) were adjusted to the LLOQ.

Publicly available data from Tijms et al. [2] were analyzed on the ADDI workbench through a virtual machine running R studio version 4.3.1. Differences between groups were calculated with Welch two-sample T-tests. Effects of baseline PKM levels on cognitive decline in preclinical AD was assessed through linear mixed-effects modeling using the *lmer*() function with results from the Mini-Mental State Exam (MMSE) and delayed recall test (Dutch version of the Rey auditory verbal learning task) adjusting for potential effects of age, sex, and level of education.

## 3 Results

### 3.1 Elevated CSF PKM levels in people with preclinical AD predicts faster cognitive decline

**Figure 1:**
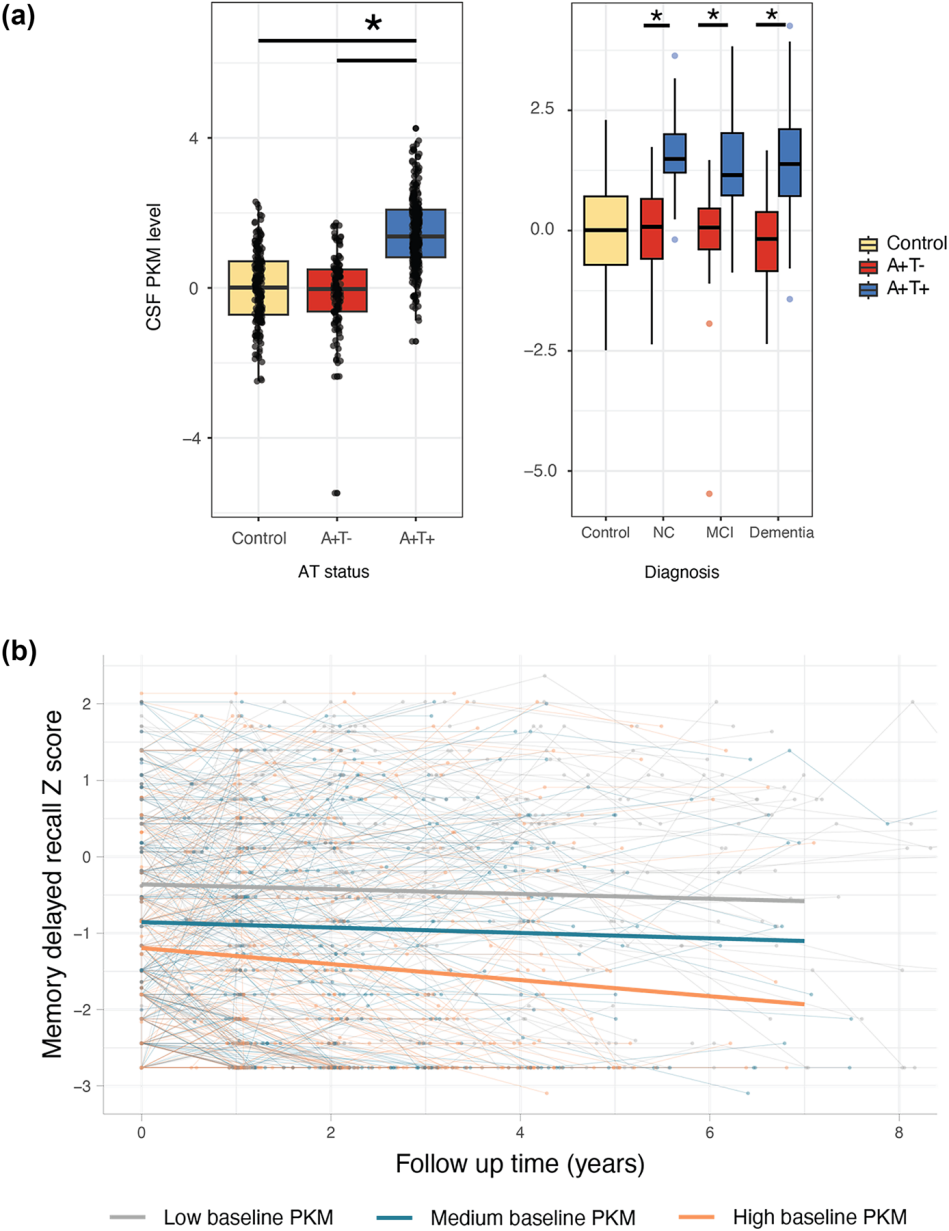
Preclinical AD patients with elevated PKM levels at baseline show accelerated cognitive decline. (a) Data from Tijms et al. [2] shows PKM levels in CSF are elevated in patients that are amyloid (A) and tau (T) positive compared to both controls and amyloid positive but tau negative patients (*p*-value = 2 × 10^−16^). This elevation in PKM was also independent of cognitive AD disease stage (NC A+T− vs A+T+ *p*-value = 4.22 × 10^−16^; MCI A+T− vs A+T+ *p*-value = 1.40 × 10^−8^; Dementia A+T− vs A+T+ *p*-value = 8.77 × 10^−16^). (b) Subjects with preclinical AD were split into three groups based on tertiles of CSF PKM abundance levels at baseline. When tracked over time the subjects with high baseline PKM levels showed a faster decline on the memory delayed recall score (linear mixed effects *p* = 0.046).

To determine whether early changes in CSF PKM levels correlate with cognitive decline in AD, we analyzed a proteomics dataset published by Tijms et al. [2], available through the ADDI workbench. This dataset contains extensive proteomics data from over 600 individuals, including control participants and AD patients with varying degrees of cognitive impairment. Control participants were defined as amyloid negative (*N* = 187). AD patients were all amyloid positive with either unimpaired cognition (NC; *N* = 107), mild-cognitive impairment (MCI; *N* = 103), or dementia (*N* = 209). Within the AD patients, 136 were negative for p-tau181 and 283 were positive for p-tau181. PKM levels were upregulated in AD patients that were positive for p-tau, compared to both p-tau negative and control participants (*p*-value = 2 × 10^−16^; Figure 1a). This upregulation of PKM by p-tau status was independent of dementia stage.

To determine whether baseline CSF PKM levels affect the rate of cognitive decline, the cohort was divided into three tertiles: high, medium, and low PKM levels at baseline. Analysis of the relationship between baseline PKM levels and MMSE over 7 years of follow-up revealed no significant association. Although MMSE is one of the most widely used tests for cognitive decline, it has been found to be less sensitive for changes in the preclinical stage of AD [22]. Alternatively, the delayed memory recall test has been shown to be more sensitive to changes in early stages of disease [23]. As we expected the changes in glycolytic metabolism to occur in the preclinical stage of disease progression, we also investigated the association between baseline PKM levels and changes in the delayed memory recall in preclinical patients (NC). A significant effect was observed, with high baseline PKM levels and associated with more rapid decline on the delayed memory recall over time (*p* = 0.04; Figure 1b). These results indicate that CSF PKM levels are elevated in patients with both amyloid and tau pathology in preclinical AD and that this elevation is associated with an accelerated cognitive decline.

### 3.2 Development and quality control of PKM ELISA

**Figure 2:**
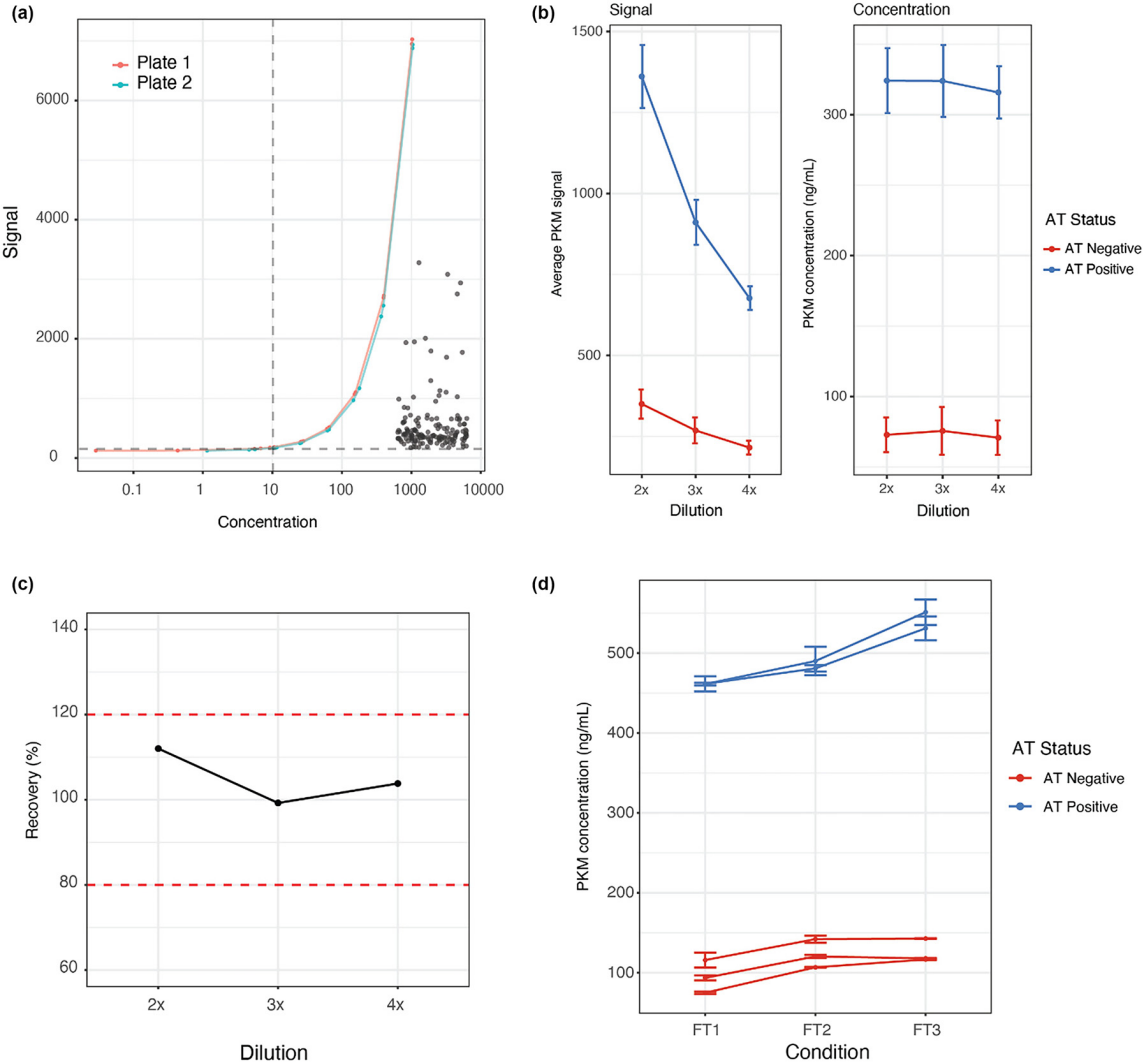
Development of MSD ELISA targeting PKM in CSF. (a) Example standard curves taken from mass spectrometry correlation experiment. Standard curve of the assay ranges from 1,000–4.1 ng/mL in dilution steps of 2.5×. Dotted line indicates the LLOQ, which was determined at 10.24 ng/mL. Grey dots indicate individual samples measured. All samples fell within the detectable range of the assay. (b) Average signal of PKM shows good dilution linearity across 2×, 3×, and 4× dilutions. The calculated concentration of PKM adjusted for the dilution factor is unchanged between the different dilutions confirming dilution linearity. Dots indicate the mean signal and concentration. Error bars indicate the standard deviation. (c) Spike recovery of PKM. Difference in average PKM signal between spiked and unspiked samples was calculated and compared to spiked diluent. Dotted lines indicate recovery ranges between 80 and 120%. (d) FT stability of PKM. Two AT positive and three AT negative samples were tested at three different FT points. No FT effect was observed for PKM.

To measure PKM levels in CSF, an ELISA was developed on the MSD platform based on two PKM targeting monoclonal antibodies. Six-fold serial dilutions of 2.5× were used to determine the assay range, which lies between 1,000 and 4.1 ng/mL (Figure 2a). The LLOQ was determined at 10.24 ng/mL, which was where the lowest standard concentration showed a duplicate CV below 20% and a recovery between 80 and 100%.

Dilution linearity was assessed using three AT positive and three AT negative samples by diluting 2× (1:1 CSF:diluent), 3× (1:2 CSF:diluent), and 4× (1:3 CSF:diluent) (Figure 2b). The average signal, unadjusted for dilution factor, dropped an average of 29.5% between each dilution for the AT positive samples and an average of 21.5% between each dilution for the AT negative samples. Average calculated PKM concentration, which is adjusted for the dilution factor, remained unchanged between dilutions, further indicating good dilution linearity.

A spike recovery experiment was performed to measure the potential interference of matrix effects (Figure 2c). Three AT positive and three AT negative samples were again diluted 2×, 3×, and 4× and each dilution was subsequently spiked with recombinant PKM to a concentration of 160 ng/mL. Percentage recovery was then determined by calculating the difference between the spiked and unspiked signal divided by a spiked diluent control. Recovery for all dilutions fell within 90–100%, representing a good recovery at these dilutions. This indicates that at these dilutions, matrix effects do not affect the measurement of PKM in CSF samples. Given the proximity of sample range at 4× dilution to the LLOQ, 3× dilution was selected for further assays.

To determine the stability of PKM over multiple freeze–thaw (FT) cycles, two AT positive and three AT negative samples were selected and each were purposefully thawed and refrozen for the experiment once (1FT), twice (2FT), and three times (3FT) (Figure 2d). No drop in concentration was observed between different FT points indicating PKM is stable over multiple FT cycles.

Interplate variability for the assay was tested by running seven identical samples across two different plates. The mean interplate CV for all samples was 5.26% and all individual interplate CVs were below 15%.

### 3.3 Correlation with mass spectrometry abundance

**Figure 3:**
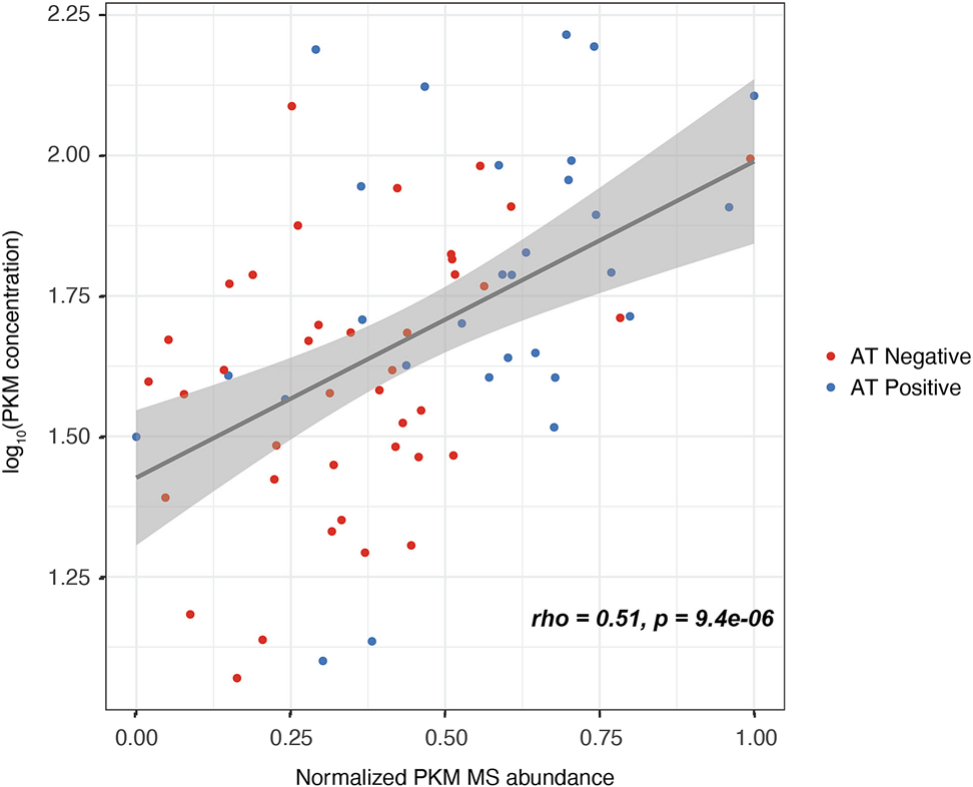
Correlation between mass spectrometry PKM abundance and ELISA concentration. A significant positive correlation was observed between the normalized PKM abundance measured by mass spectrometry and logged PKM concentration measured by MSD ELISA (spearman rho = 0.51, *p* = 9.4 × 10^−6^). Red dots indicate AT negative participants and blue dots indicate AT positive participants.

To determine the degree to which measurements of the newly developed ELISA agree with mass spectrometry measurements, we quantified PKM in a set of 69 CSF samples that was previously analyzed using data-independent mass spectrometry [10]. This set comprised 40 AT negative and 29 AT positive patients. All samples were measured with a duplicate CV below 20% with an average of 4.23%. Normalized PKM abundance measured with mass spectrometry was correlated with the logged PKM concentration measured through ELISA (Figure 3). A significant positive correlation was observed (Spearman rho = 0.51, *p*-value = 9.4 × 10^−6^), indicating a reasonable strength agreement between the two methods.

### 3.4 Validation in independent cohort

**Figure 4:**
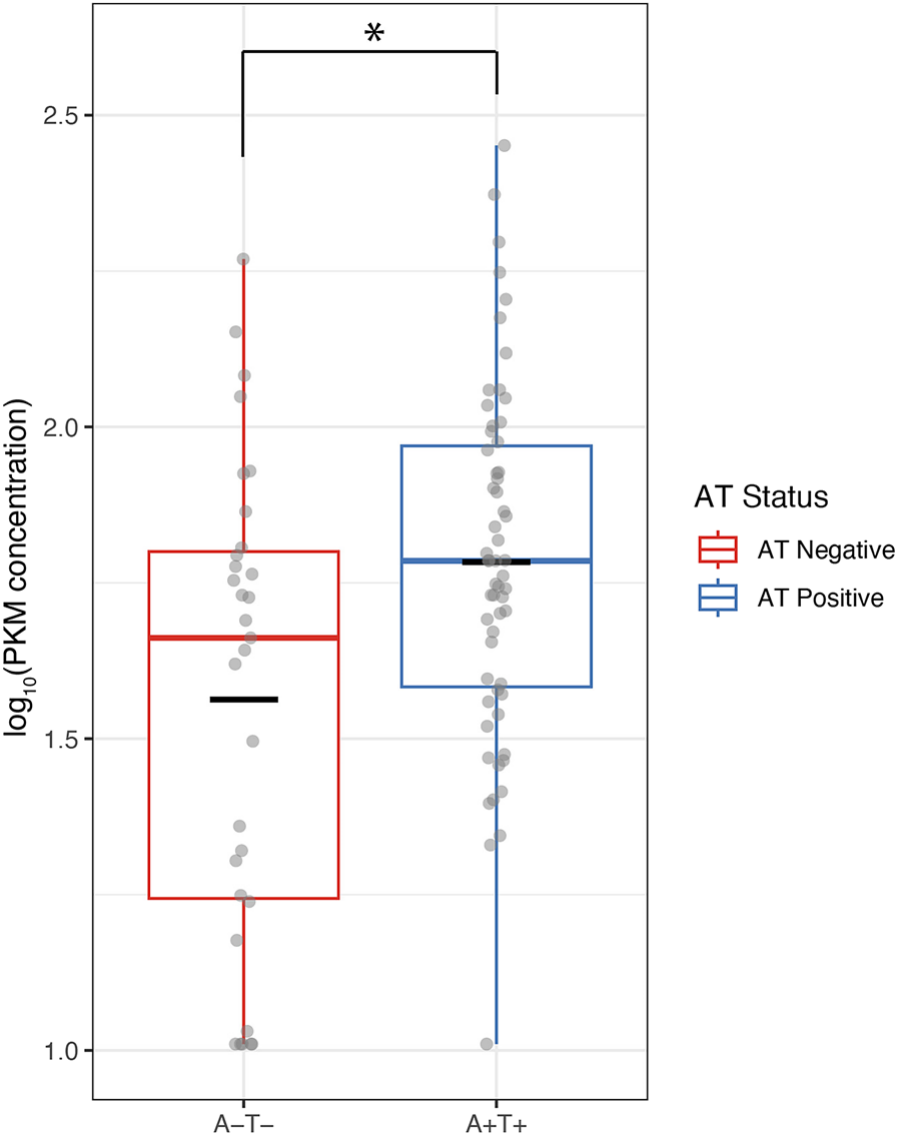
Validation of PKM assay on an independent cohort. 31 AT negative and 59 AT positive samples were measured. PKM was found to be elevated in the AT positive group compared to the AT negative group (*p*-value = 0.003). Boxplots indicate median and interquartile range. Black line indicates the mean and grey dots show the individual sample measurements.

We tested the newly developed assay in an independent set of samples not previously tested by mass spectrometry to validate the upregulation of PKM in AT positive patients. A total of 90 samples were tested, consisting of 59 AT positive and 31 AT negative samples. Six (5 AT− and 1 AT+) samples had a calculated concentration that fell below the LLOQ of the assay, which were replaced with the LLOQ concentration of 10.24 ng/mL for further analyses. PKM levels were found to be elevated in the CSF of AT positive participants compared to AT negative participants (Figure 4; *p*-value = 0.003).

## 4 Discussion

Neuroimaging studies have suggested brain glucose metabolism to be one of the earliest affected aspects in AD, as indicated by a reduced glucose uptake in the brain observed with FDG-PET [8,24]. Alternatively, proteomics studies in CSF have pointed toward an upregulation of glycolytic enzymes [10,12,13]. How the changes in glycolytic enzymes in CSF relate to the observed reduction in glucose uptake in the brain and to the other pathologies over the course of disease progression remains unclear. The ability to detect changes in key regulating enzymes of this process could improve stratification of AD subpopulations and facilitate tracking disease progression, especially in the early stages of the disease. As one of the key regulators of cellular glucose metabolism PKM is a prime candidate for monitoring these metabolic changes and has previously been proposed as a candidate biomarker for AD [10,13].

Our observation here of increased PKM levels in CSF in a tau-dependent manner indicates a potential interplay between tau pathology and brain metabolic changes. This is in line with previous studies that have indicated a link between tau pathology and metabolic dysfunction [25,26]. Specifically, Adams et al. [25] showed, through neuroimaging of both glucose consumption and tau deposition, that regions with high tau deposition exhibited hypometabolism of glucose. The effects we observed between higher PKM levels and faster cognitive decline in preclinical patients further supports the notion that glycolytic dysfunction occurs early in the disease process. Chronologically, we hypothesize that glycolytic dysfunction and tau deposition coincide. This agrees with previous studies using mass spectrometry that also showed levels of PKM to increase concurrently with tau levels in CSF [4,27]. It remains to be determined, however, whether metabolic changes permit the spreading of tau tangles or whether tau deposition induces a shift in glucose metabolism.

In the present study, we developed a novel immunoassay to accurately quantify levels of PKM protein in CSF. First, using publicly available proteomics data, we showed that levels of PKM are increased in AD CSF in a tau-dependent manner and that these higher levels correlate with faster cognitive decline in preclinical AD patients. We tested several commercially available chemiluminescent ELISA kits for PKM, but did not find a kit that had sufficient sensitivity to quantify PKM in CSF. We therefore developed an electrochemiluminescence-based ELISA (MSD) to provide the sensitivity to quantify changes in PKM levels in CSF. Through various quality control steps, we showed that this assay can robustly quantify PKM protein in human CSF samples. While a small number of samples fell below the LLOQ, and were subsequently replaced by the LLOQ detection, we do not expect these results to significantly impact the findings of our study, as our focus was on the elevation of PKM. Concentration measurements from the ELISA had a reasonable and significant, but not excellent, correlation with mass spectrometry measurements in matching samples. The antibodies used in our assay were not specific to one of the isoforms of PKM1/2, and it is possible the assay interacts differently with the two isoforms. Alternatively, certain proteoforms of PKM could exist in the CSF that are picked up by mass spectrometry but not by our ELISA. Although we have previously shown that the most abundant species of PKM in CSF is full-length [10], it is possible that some of the unexplained variability between the mass spectrometry and ELISA measurements could arise from this. Another potential source of variation between the methods could come from sample preparation. In mass spectrometry total protein levels in samples are normalized between samples, which is not usually done in ELISA methods. Finally, we tested the assay on a new set of samples, confirming elevation of PKM levels in AT positive subjects.

Various cellular processes could be the cause for an increased energy demand in the brain, such as elevated neuronal activity or reactive gliosis from microglia and astrocytes. A snRNA-seq study revealed transcriptional elevations in glycolytic proteins, including PKM, in excitatory neurons and astrocytes in AD [28], but it remains unclear what the source of elevated PKM in CSF is. Neuronal hyperactivity in hippocampal as well as cortical regions has been consistently observed in the early stages of AD [29]. Furthermore, one recent *in vitro* study found that soluble tau in CSF taken from AD patients can induce hyperexcitability in *ex vivo* mouse brain slices leading to neuronal hyperactivity [30]. Neuronal hyperactivity in the early stages of disease shifts to hypoactivity in later stages, which has also been tied to tau-dependent neuronal loss [31,32]. Apart from neurons, glial cells are a major consumer of brain glucose and are crucial in maintaining brain homeostasis. Both astrocytes and microglia are involved in the neuroinflammatory process in response to neurodegeneration in AD [33–35]. A recent study, using large-scale single-cell transcriptomics on post-mortem brain tissue found that glial cells had increased expression of glycolytic genes, including PKM, across different brain regions at different stages of AD [17].

Data from Johnson et al. [4] suggested that over the course of AD development there are two events of elevated glycolytic metabolism. We hypothesize that the early elevation, coinciding with the start of tau deposition, is tied to the potential tau-induced neuronal hyperactivity. Then, the second elevated glycolytic event, coinciding with clinical onset and neuronal loss, is possibly derived from the reactive gliosis where microglia and astrocytes respond to the neuronal injury. The data from Johnson et al. [4] also suggest that the second glycolytic elevation event is more specific to the PKM isoform, PKM2, and coincides with the start of neuronal inflammation. Previous studies have suggested varying functions of the PKM isoforms in both AD and cancer [19,36]. Traxler et al. [19] also showed that PKM2 interacts with HIF1, a hypoxia associated transcription factor, promoting neuronal loss, possibly indicating that the second peak of PKM elevation is hypoxia related. One recent study showed that PKM2 specifically mediated microglia activation in an epilepsy mouse-model [37]. Another study in an ischemia mouse-model showed that a PKM2 knockout in astrocytes increased neuronal damage, which could be reversed by supplemental lactate [38].

### 4.1 Limitations

A limitation of the novel assay we developed here is that it is not able to discern the different isoforms of PKM, derived through alternative splicing of two exons [39], and rather measures PKM1/2 levels in CSF. To further study the specific contribution of PKM2 vs total PKM1/2 over the course of disease progression, the assay could be readily adapted by exchanging one of the antibodies for a PKM2 specific antibody. Additionally, although suggesting an interplay between tau deposition and metabolic changes, this study was limited in further determining the cause vs consequence. Application of this assay to a cohort with serial sample collections over the course of disease onset is necessary to determine the temporal dynamics between tau pathology and metabolic changes.

Our study provides a novel assay to measure the PKM levels in CSF. Further validation in other cohorts with serial measurements over disease progression and with matching cognitive test measures will provide insights in the direct clinical potential of this novel assay. While not intended to replace the Amyloid, Tau, Neurodegeneration framework for AD diagnosis, we suggest that elevated PKM levels could indicate a more vulnerable group of patients in the early stages of AD, driven by a shift in glycolytic metabolism. This assay could be used to further test this hypothesis, addressing the heterogeneity of the disease. In conclusion, this study provides a research tool that can be used to study the dysregulation of glucose metabolism over the course of disease development and elucidate the timing of the relationship between tau pathology and glucose metabolism in AD.

## Supplementary Material

Supplementary Table
